# Bacterial magnetic particles improve testes-mediated transgene efficiency in mice

**DOI:** 10.1080/10717544.2017.1293195

**Published:** 2017-03-10

**Authors:** Chao Wang, Guanghong Sun, Ye Wang, Nana Kong, Yafei Chi, Leilei Yang, Qiliang Xin, Zhen Teng, Xu Wang, Yujun Wen, Ying Li, Guoliang Xia

**Affiliations:** 1 State Key Laboratories for Agrobiotechnology and College of Biological Sciences, China Agricultural University, Beijing, China,; 2 School of Basic Medical Science, Capital Medical University, Beijing, China,; 3 Department of Pathology, Eye Hospital of Hebei Province, Hebei, China, and; 4 Ningxia Key Laboratory of Cerebrocranial Diseases, Department of Anatomy, Histology and Embryology, School of Basic Medical Sciences, Ningxia Medical University, Yinchuan, China

**Keywords:** Testis, transgene, mice, bacterial magnetic particle, efficient

## Abstract

Nano-scaled materials have been proved to be ideal DNA carriers for transgene. Bacterial magnetic particles (BMPs) help to reduce the toxicity of polyethylenimine (PEI), an efficient gene-transferring agent, and assist tissue transgene *ex vivo*. Here, the effectiveness of the BMP-PEI complex-conjugated foreign DNAs (BPDs) in promoting testes-mediated gene transfer (TMGT) in mouse was compared with that of liposome-conjugated foreign DNAs. The results proved that through testes injection, the clusters of BPDs successfully reached the cytoplasm and the nuclear of spermatogenesis cell, and expressed in testes of transgene founder mice. Additionally, the ratio of founder mice obtained from BPDs (88%) is about 3 times higher than the control (25%) (*p* < 0.05). Interestingly, the motility of sperms recovered from epididymis of the founder mice from BPD group were significantly improved, as compared with the control (*p* < 0.01). Based on classic breeding, the ratio of transgene mice within the first filial was significantly higher in BPDs compared with the control (73.8% versus 11.6%, *p* < 0.05). TMGT in this study did not produce visible histological changes in the testis. In conclusion, nano-scaled BPDs could be an alternative strategy for efficiently producing transgene mice *in vivo*.

## Introduction

Genetically modified laboratory animal models are indispensable for biomedical researches. However, the most popular methods in producing such animals remains low efficiency, which either requires high technologies, expensive instruments, has safety risks or brings adverse effects upon cells and tissues (Smith, 2004; Parrington et al., 2011), and as a result, are not easily handled by individual laboratories. Previously developed spermatozoa-based gene transfer methods, including testis- and sperm-mediated gene transfer (TMGT and SMGT) can be performed by general researchers to achieve personalized gene modifications in animals (Coward et al., 2007; Collares et al., 2010; Amaral et al., 2011; Campos et al., 2011a,b). Referring to TMGT, foreign DNA vehicles need to be excellent in penetrating thick tissues to achieve better output. Besides, although the uptake of foreign DNA by target cells could be improved through either “*in vivo*” electroporation or pre-hatching the DNA solution with materials such as liposome, calcium phosphate, and DMSO (Sato et al., 2002; Coward et al., 2007; Campos et al., 2011b), the output remains disappointed due to the poor DNA integration ratio and cytotoxicity of the mediators (Sato et al., 2002; Hooley et al., 2009). Alternatively, cationic macromolecular polymers, such as polyethylenimine (PEI), are attractive non-viral molecules for high transferring efficacy of nucleotides (Read et al., 2010; Ma et al., 2011; Marszall, 2011; Lee et al., 2012; Mandal et al., 2013). However, the cell toxicity of the material hinders its wide application for years as well (Chollet et al., 2002; Tong et al., 2005). Therefore, it is necessary to renovate low technique required gene delivery strategies that employs both cheaper and safer materials and has high efficiency (Coward et al., 2007; Villemejane & Mir, 2009; Patnaik et al., 2010; Parrington et al., 2011). From our point of view, the key point is to promote foreign DNAs diffusion across the testis interstitial tissue and improve the absorption by germ cells with reduced toxicity based on PEI.

Applying magnetic nanoparticle materials in biomedical science offers major advantages by their unique size and physicochemical properties. Of which, iron oxide nanoparticles, the magnetite (Fe_3_O_4_) are by far the most commonly employed particles for biomedical applications (Akbarzadeh et al., 2012). Bacterial magnetic particles (BMPs) are comprised by Fe_3_O_4_ particles of 45–55 nm in diameter and are enveloped by cytoplasmic membrane, which presents a friendly surface for the particles to penetrate the bio-membrane (Xie et al., 2009; Marszall, 2011). For instance, BMP-carrier gene vaccine plus a magnetic field results in tumor protection (Tang et al., 2012; Stanley et al., 2015). BMPs have been used as the carriers for nucleic acids (Ota et al., 2003) and have advantages over artificial iron oxide nanoparticles in that they can not only be cheaply produced and safely sterilized (Xiang et al., 2007), but that they can easily couple with bioactive macromolecules, anticancer drugs, or liposomes (Matsunaga et al., 2007; Rieck et al., 2013). Interestingly, combining BMPs to PEIs proves to be helpful for reducing the cytotoxicity of PEI *in vitro* and *in vivo* (Marszall, 2011; Zuo et al., 2012), but whether BMP-PEI complex can be used in TMGT or SMGT remains unclear.

Consequently, it is our interest to clarify if BMP-PEI complex-conjugated foreign DNAs (BPDs) can be used in TMGT as well. This study is then designed to clarify the effect of BPDs on improving TMGT in mice. The results showed that nano-scaled BPDs could be an alternative strategy for efficiently producing transgene mice *in vivo*.

## Materials and methods

### Chemicals and animals

All chemicals and reagents were purchased from Sigma-Aldrich Inc. (St. Louis. MO) unless otherwise indicated. DNA solution and injection buffer was purchased from Chemicon International (Merck KGaA, Darmstadt, Germany). Mouse embryo culture media, such as M2, M2 supplemented with hyaluronidase and M16 were purchased from Millipore (Billerica, MA). Equine chorionic gonadotrophin (eCG) was purchased from Ningbo Hormone Product Inc. (Ningbo, ZheJiang, China). PEI (25 k Da, pH 7.2) was diluted with de-ionized water to the working concentration of 2 μg/μl and stored at 4 °C until use.

Sexually matured (over 4-week-old female and 4-month-old male) FVB/N mice, and outbred ICR mice with normal fertility from the Academy of Military Medical Sciences, Beijing, China, were used for all TMGT experiments. Mice were kept under barrier housing facilities with controlled temperature (24–26 °C) and light (12 h light and 12 h dark cycles), with food and water *ad libitum*. Briefly, for intra-testicular injection, 24 male FVB/N mice were prepared. The experiments were carried out in accordance with the principles and guidelines for the use of laboratory animals and approved by Institutional Animal Care and Use Committee of the China Agricultural University.

### BMPs preparation

The production and purification of BMPs were performed according to the previous report (Sun et al., 2008). In brief, the *Magnetospirillum gryphiswaldense* MSR-1 cells (German Collection of Microorganisms and Cell Cultures, Germany), which is characterized with its ability to accumulate BMPs within the cytoplasm, were submerged, cultured, and then re-suspended in 10 ml of 5 mM phosphate-buffered saline (PBS, pH 7.4) and disrupted by two passes through a French press at 1000 psi (Thermo Electron Corp., Waltham, MA, USA). After examining disrupted cells under an optical microscope, BMPs were collected using neodymium-iron-boron magnets (Nd-Fe-B magnet ⊄ 29 × 8 mm), which were produced by a non-homogeneous magnetic field (0.5 T at the surface). After removing the supernatant, the BMPs were washed 8–10 times with PBS using a mild ultrasonic bath (at 50 W). All of the BMPs were lyophilized by freeze/drying for 20 h, then sterilized by γ-rays (15 k Gy), and stored at −20 °C until further use. Whenever use, the magnetosomes were diluted with PBS (pH 7.4) at a concentration of 10 ng/μl and stored at 4 °C until use.

### DNA preparation

**Figure 1.**
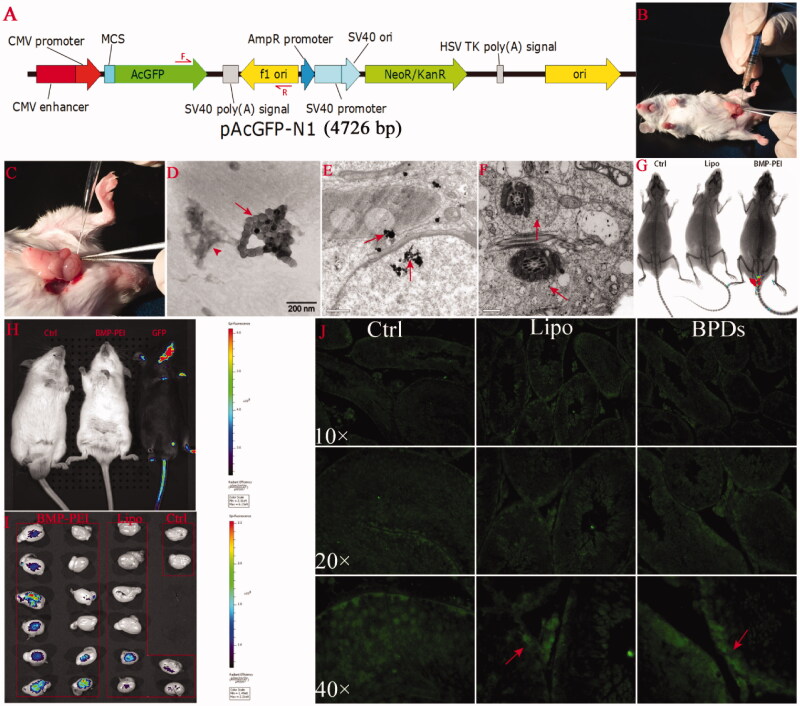
BPD preparation, intra-testicular injection, and foreign gene expression after TMGT in the testes. (A) The structure of pAcGFP-N1 plasmid as well as the loci of primers (red arrows) designed for PCR procedure. (B, C) Surgical procedures for intra-testicular injection, in which a 27-gauge needle is used to pierce the tunica albuginea of the testis at one of the ends of the long axis to assist penetration of mouth pipette into the tissue (B). Mouth pipette was withdrawn from the testis bit by bit while solution was injected into the testes (C). (D) The clusters of BMPs (red arrow) that have conjugated with PEI-DNAs (red arrow head) observed under TEM. The size distribution of BMPs ranged from 45 to 55 nm. Amplification: × 60 000. (E, F) BPDs were presented within the cells of seminiferous tubule (red arrows). BPDs were observed both within the cytoplasm as well as nuclear of spermatogenic cells (E, bar = 200 nm) and within the tails of sperms (F, bar = 500 nm). (G, H, I) *In vivo* and *ex vivo* GFP fluorescence detection. (G) The fluorescence of GFP protein was presented in the testes of BMP-PEI group mouse under KODAK Image Station *In-Vivo* FX system. (H) No fluorescence was observed in the testes of mice coming from both groups, as compared with the GFP transgene positive mice obtained through conventional pronuclear injection method (black mouse on the right), but can be detected under IVIS QUANTUM FX system when mouse testes were recovered. (J) The expression of GFP protein was shown in testes injected with BPDs and Lipo-DNAs (red arrow) under fluorescence microscope.

The pAcGFP-N1 vector coding for green fluorescent protein (GFP) under the control of the cytomegalovirus (CMV) promoter as a reporter gene was used for transgene assays (Figure 1A). Briefly, the plasmid purchased from CLONTECH Laboratories Inc. (Mountain View, CA, USA). Purified plasmids with a ratio of OD 260/280 over 1.8 was used. The digested 4726 bp DNA fragment was re-suspended in Tris (1 mM) – EDTA (0.1 mM) buffer (pH 7.4). The store concentration was 1 μg/μl.

### BMP-PEI-DNA complexes (BPDs) preparation

BPDs were prepared according to previous description (Xiang et al., 2007; Yang et al., 2016). In brief, the ratio among BMPs, PEI and DNA in the complex was fixed to 1:1.6:0.8 (μg/μg/μg). For each testis, immediately before injection, 8 μl (1 μg/μl) of foreign DNA solution for every testis was mixed with 8 μl PEI (2 μg/μl) and incubated for 10 min to form PBDs. Meanwhile, in order to thoroughly re-suspend the magnetosomes, the BMPs solution tube was firstly incubated in a mild ultrasonic bath (at 50 W) for 10–20 s and then immediately put on the ice for cooling, the procedure was repeated 3–4 times until the suspension turned into brown color and looked like half transparency. Then, quickly took 10 μl of BMPs (1 μg/μl) suspension and mixed with PEI-DNA solution mildly with the pipette for 10 s, and incubated the tube for 10 min for self-assemble.

Foreign plasmid pAcGFP-N1 mixed with liposome (lab made and commonly used) was used as the control (Lipo-DNAs in short). The mixture was prepared according to the manufacturer’s instruction. In brief, diluted 1 μl (1 μg/μl) of DNA in 50 μl of PBS (pH 7.4), mixed gently before incubating with liposome. Mixed liposome store solution gently before use, then diluted 3 μl liposome in 50 μl of PBS (pH 7.4), incubated for 5 min at room temperature. Then combined the diluted DNA and diluted liposome solution, mixed gently and incubated for 20 min at room temperature before use.

Whenever performing TMGT, 12 μl of either BPDs, Lipo-DNAs, or PBS (black control) was filled into a capacity sterile mouth pipette of 40 μm in diameter for testis injection assay.

For observing images of formed PBDs complexes under transmission electron microscopy (Tecnai G2 F30, FEI, OR, USA), 20 μl of the complexes were applied to the copper grids at an accelerating voltage of 300 kV.

### Testes injection of BPDs

For TMGT experiments, mice were randomly grouped and respectively injected with either BMP-PEI-DNA complexes (BPDs) (19 mice), Lipo-DNA complexes (12 mice) (control), or PBS (3 mice) (pH 7.4) (blank control). The surgical operation protocol was as following. Mice were anesthetized with 40 mg/kg BW of 1% sodium pentobarbital. First, each testis of the individual mouse was gently pulled out from the celiac surgically. Before injection, carefully pierce the tunica albuginea of the testis at one of the ends of the long axis with a 27-gauge needle to assist penetration of mouth pipette into the tissue (Figure 1B). Then mouse pipette was plugged into 2/3 depth of the testis along the long axis through the wound. The suspension of the DNA complex was injected into the testis bit by bit while slowly withdrawing the pipette (Figure 1C). The injected volume was about 20–30 μl for each testis. After injection, the testis was returned into the celiac gently. After the other side of the testis injection was accomplished, the wound was sutured. Later, the mice were put onto a warm bed (25 °C) in supine position and a neodymium-iron-boron magnet with an intensity of 600 mT (Tang et al., 2012) was placed onto the surface of the abdomen until the mice recovered (5–10 min after operation).

### *In vivo* and *ex vivo* examinations of transgene effect

For *ex vivo* examination, 3 days after surgery, the foreign DNA integration and expression effect in the testes of mice (6 from BPD group, 4 from Lipo-DNA group, and 1 from PBS group) undertaken TMGT were sacrificed. The testes of each mouse were immediately recovered. The GFP protein expression within the testes were examined through IVIS QUANTUM FX system (PerkinElmer, Waltham, MA, USA) according to the instructions of the producer.

For *in vivo* examination, 7 days after surgery, the foreign DNA integration and expression effect in the testes of mice undertaken TMGT were determined through a KODAK Image Station *In-Vivo* FX system (Eastman Kodak Company, Rochester, NY). In brief, mice (6 from BPD group, 4 from Lipo-DNA group, and 2 from PBS group) were anesthetized and denuded with 8% Na_2_S plus 30% ethanol in PBS solution. The examinations were immediately performed according to the instructions of the producer.

### BMP particles detection through TEM in founder mice

To justify if BMPs are present inside the spermatogenic cells in TMGT mice, 7 days after surgery, the testes of the founder mice were sliced and observed under TEM (JEM 2100, Electronics Co., Ltd., Japan). Briefly, the testes were fixed with 2% glutaraldehyde and 1% osmium tetroxide, and then sliced and observed under TEM according to the instructions of the producer.

### Histochemistry

Thirty days after TMGT, the influence of surgical injection of foreign solutions on testes histological structure of the founder mice was evaluated. For histological examination, the testes of mice injected with BPDs and PBS were fixed, embedded in paraffin, and sectioned to a thickness of 5 μm. After de-waxing, rehydration, the sections were dyed with hematoxylin-eosin staining. The structures of testes were examined under a microscope (Eclipse, Nikon, Japan).

### Immunofluorescence

Twenty-five hours after TMGT, the influence of surgical injection of foreign solutions on testes histological structure of the founder mice was evaluated through immunofluorescence examination. Briefly, the testes of mice injected with BPDs and PBS were fixed, embedded in paraffin, and sectioned to a thickness of 5 μm. After de-waxing, rehydration, microwave antigen repair and sealed by serum, the sections were dyed with propidium iodide solution (BioLegend, San Diego, CA, USA) for nucleus and Chk pAb to GFP (Abcam, Cambridge, UK) for GFP protein. The structures and fluorescence of testes were examined under a microscope (Eclipse, Nikon, Japan).

### Spermatozoa motility analyses

The influence of BPDs and Lipo-DNAs on spermatozoa motility in epididymis of the founder mice was evaluated according to previous reports (Bian et al., 2012). In brief, 30 days after TMGT, spermatozoa from vas deference and caudal epididymis of TMGT mice were cultured with mT6 medium at a 37.8 °C incubator under 5% CO_2_ for 1.5 h to capacitate *in vitro*. For spermatozoa motility analysis, a CASA system (Version 12 CEROS, Hamilton Thorne Research, Beverly, MA) was used with the following settings: for cell detection: minimal contrast, 50; minimal cell size, 4 pixels; and 60 frames were acquired at a frame rate of 60 Hz. At least 100 tracks were measured for each sample at 37.8 °C with a Slide Warmer (#720230, Hamilton Thorne Research, Beverly, MA). The measured kinetic parameters were: VAP, average path velocity; VCL, curvilinear velocity; VSL, straight line velocity; ALH, amplitude of lateral head displacement; BCF, beat cross frequency; LIN, percentage of linearity (VSL/VCL 100%); STR, percentage of straightness.

### Polymerase chain reaction (PCR) procedure

To detect the integration ratio of foreign DNAs in all candidate transgene positive mice, the DNAs from the tail tissue of each mouse mice of the first generation of all founders were extracted by using phenol-chloroform extraction and ethanol precipitation method, as has been reported previously (Zuo et al., 2012). The samples were then quantified by measuring the A260/A280 value through a microplate absorbance reader (Bio-Rad 680, Hercules, CA, USA). And the quality of each sample was evaluated by agarose gel electrophoresis. Then, the samples were diluted to 100 ng/μl and stored at −20 °C for later use as templates in PCR.

Each 25 μl PCR amplification system contained the following reagents: 12.5 μl 2 × Mix (Zomanbio, Beijing, China), 9.5 μl dH_2_O, 0.5 μM of each GFP specific primer (Shanghai Bio-Engineering Co., Shanghai, China), and 2 μl template DNA. PCR protocol was: pre-denaturation at 95 °C for 5 min; 35 cycles of denaturation at 95 °C for 30 s, annealing at 60 °C for 30 s, and extension at 72 °C for 30 s; and a final extension at 72 °C for 5 min. The sequences of the GFP primers were: forward: 5′-TACCCTGGTGAATCGCATCG-3′ and reverse: 5′-TTTCGCCCTTTGACGTTGGA-3′. These primers amplified a 750 bp sequence of the GFP gene. PCR was performed with a Bio-Rad thermocycler (Hercules, CA, USA), and the products were subjected to electrophoresis in a 1.5% agarose gel. The band signals were acquired using Infinity 3026 analysis software. The GFP PCR amplicons were sequenced to confirm that they were identical to the gene sequences. The PCR procedure was double checked in 2 weeks by collecting fresh samples.

### Data analysis

Data were analyzed either by *t*-test and ANOVA (for analysis of sperm motility data only). Values of *p *< 0.05 were considered statistically significant.

## Results

### BMP conjugated successfully with PEI-DNA complex

With the help of TEM detection, BMP particles were proved to be able to conjugate with PEI-DNA complexes successfully. As shown in Figure 1(D), the diameter of each BMP was approximately 45–55 nm size. In PBS buffer, BMP particles, PEI and DNAs conjugated to each other excellently, where the BMP particles generally formed into clusters (red arrow), and the PEI-DNA complexes conjugated to the cluster of BMPs (red arrow head). The results indicated that BMP cluster could conjugate with PEI-DNA complex steadily.

### BPDs can get into nuclear of spermatogenic cells

As was demonstrated by TEM observation of BPDs-injected testes tissue, in seminiferous tubule, the presentation of BPDs within the cytoplasm as well as nuclear of spermatogenic cells were detected (Figure 1E, red arrows). In addition, the BPD clusters were detected in the cytoplasm of sperm tails as well (Figure 1F). The results imply that BPDs could penetrate the membranes of a cell and reach the chromosomes of spermatogenesis cells easily.

### Transgene was expressed in testes of both BPD and Lipo-DNA groups

In order to evaluate if foreign DNAs could be expressed within the testes, the GFP proteins within testes of both BPD and Lipo-DNA group mice were detected through both *in vivo* (Figure 1G and H) and *ex vivo* (Figure 1I) live image systems. Under KODAK Image Station *In-Vivo* FX system, the GFP expression was observed while the testes were intact within denuded mice body (Figure 1G). On the other hand, under, IVIS QUANTUM FX system, although GFP expression could not be detected while the testes were intact within the mice, as compared with the GFP transgene positive mice obtained through conventional pronuclear injection method (black mouse on the right) (Figure 1H), the GFP fluorescence was observed after testes were recovered from the body (Figure 1I). Meanwhile, the GFP protein was detected in testes 25 h after injection in both groups except control (Figure 1J). The results showed that the sensitivity of the live fluorescence image in the two systems have respective characters. The GFP protein could express in testes of both BPD- as well as Lipo-DNA-injected mice.

### BPDs is superior to Lipo-DNAs in preserving sperm motility

**Figure 2.**
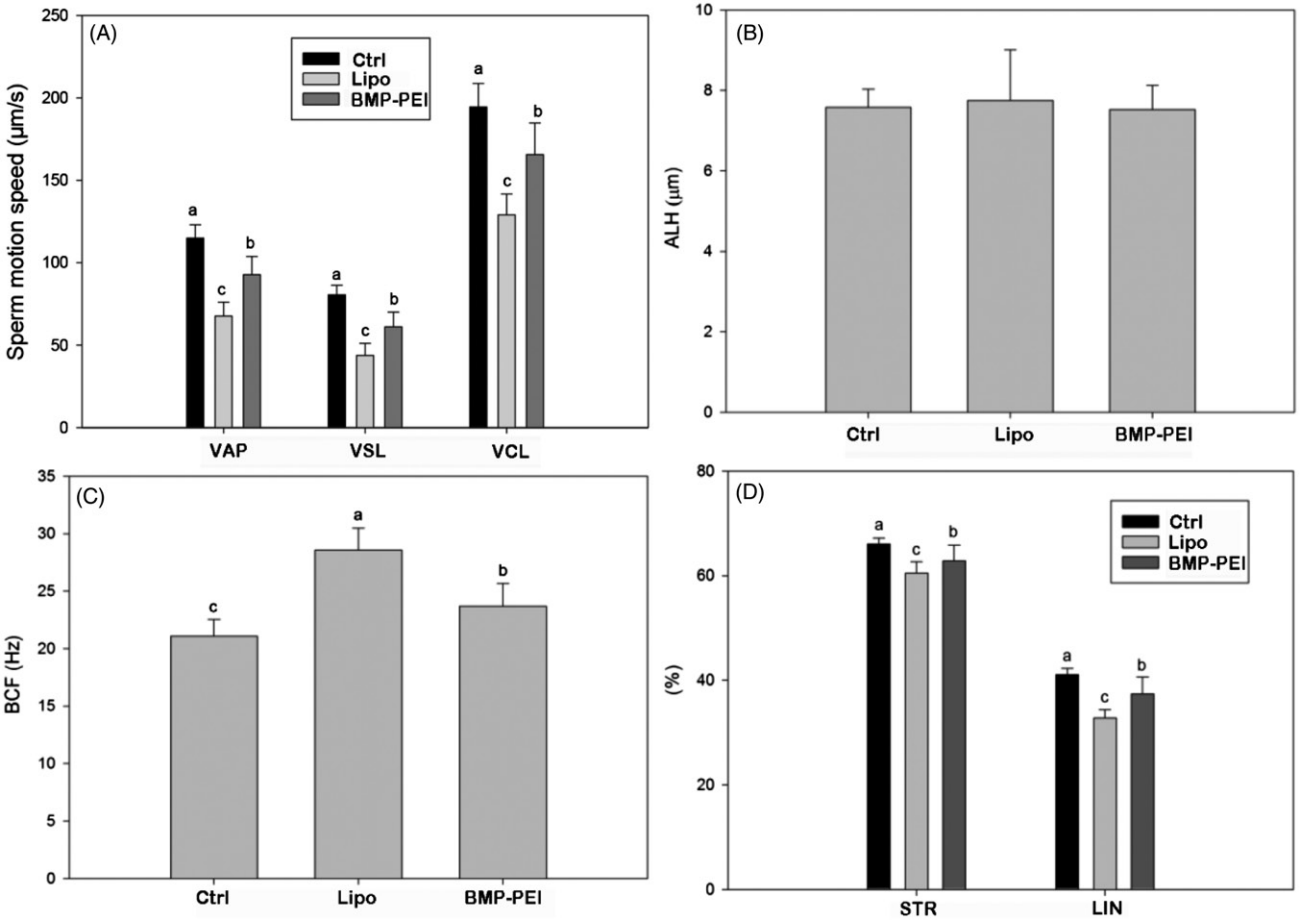
The influence of different DNA carriers on sperm motility after TMGT. From chart A through D, different letters in each chart indicate significant differences among groups (ANOVA, *p* < 0.05). As can be seen, almost all sperm motility parameters were significantly different among BPDs, Lipo-DNAs, and the controls except ALH (B). In addition, the increase of VAP, VSL, VCL, LIN, and STR in BPD groups, which represents the improved sperm motility, was significantly higher than the Lipo-DNA groups.

In order to evaluate the toxicity of BMP-PEI-mediated gene modification compared to commercial liposome products, the sperm mobility within epididymic ducts of TMGT mice were examined. In this aspect, multiple parameters for judging the motility of sperms were considered, including curvilinear velocity (VCL), straight-line velocity (VSL), average path velocity (VAP), amplitude of lateral head displacement (ALH), beat cross frequency (BCF), percentage of linearity (LIN = VSL/VCL × 100%), and percentage of straightness (STR = VSL/VAP × 100%). The results showed that almost all of the parameters of BPD groups, except ALH, were most significantly higher than those in Lipo-DNAs and controls (*p* < 0.01) (Figure 2). The results approved the fact that BPDs have less toxicity over Lipo-DNAs in preserving sperm motility.

### Transgenic efficiency in BPD group is higher than Lipo-DNA group

**Figure 3.**
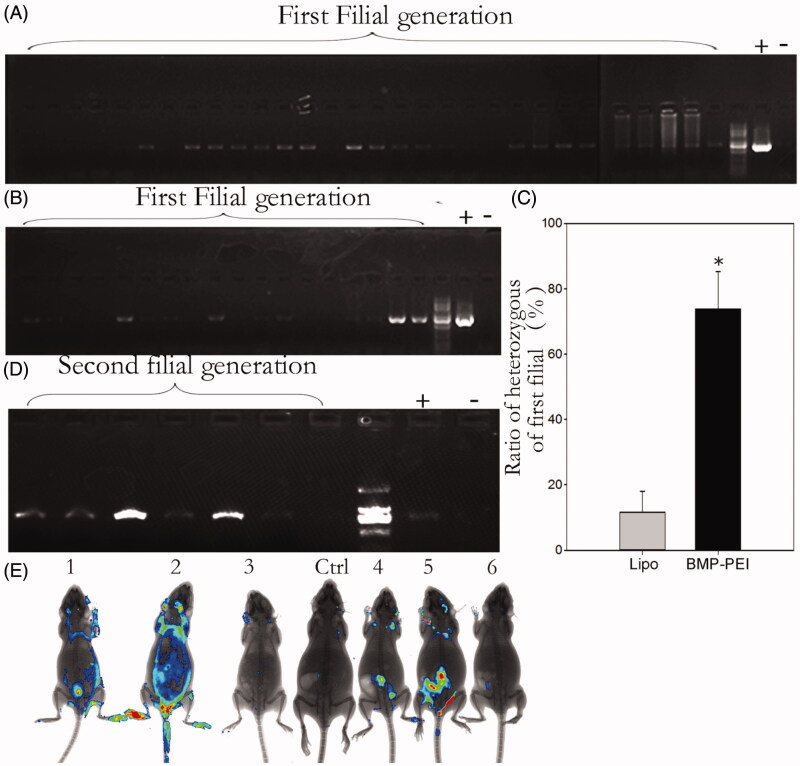
Genotype analysis of the first- and the second generations of founder mice derived from TMGT. (A, B) Demonstration of PCR analysis of plasmid encoding *GFP* integration in the chromosomes of offspring derived from some of the founder mice tail tissue. A: BPDs; B: liposome. (C) The ratios of heterozygous in the first filial between BPD and Lipo-DNA treatments, of which the ratio in BPDs was 6 times higher than the Lipo-DNA group (73.8% versus 11.6%, *p* < 0.05). (D) Demonstration of the genotypes of the second generation of founder mice after BPD injection. It showed that transgene can be successfully inherited by the second filial after inbreeding of the first filial. (E) Demonstration of the transgene that was expressed in some of the tissues of the second filial bodies after inbreeding of the first filials, as was approved by Kodak Image Station In-Vivo FX system. These transgene mice came from the founder mice No. 1.

In order to demonstrate that the injected GFP plasmids could integrate into sperm chromosomes and be passed to the filial, the founder mice of both groups were mated to respective wild type mice. Then the genotypes of the filials of the first generations were examined through PCR procedures (Figure 3). The results showed that both BPDs and Lipo-DNAs could be passed to the offspring of the founders (Figure 3A and B). However, the transgenic heterozygous mice obtained from BPDs were almost 6 times higher than the Lipo-DNA treatment (73.8% versus 11.6%, *p* < 0.05) (Figure 3C). When the heterozygous of the BPD group-derived mice were inbred to each other, transgene homozygous mice were obtained, as PCR results showed (Figure 3D). In addition to that, the GFP fluorescence was observed under KODAK system as well (Figure 3E). Therefore, although the heredity of transgene is not 100%, both BMP-PEI- and liposome-conjugated DNAs could be passed from founder mice to their offspring.

**Figure 4.**
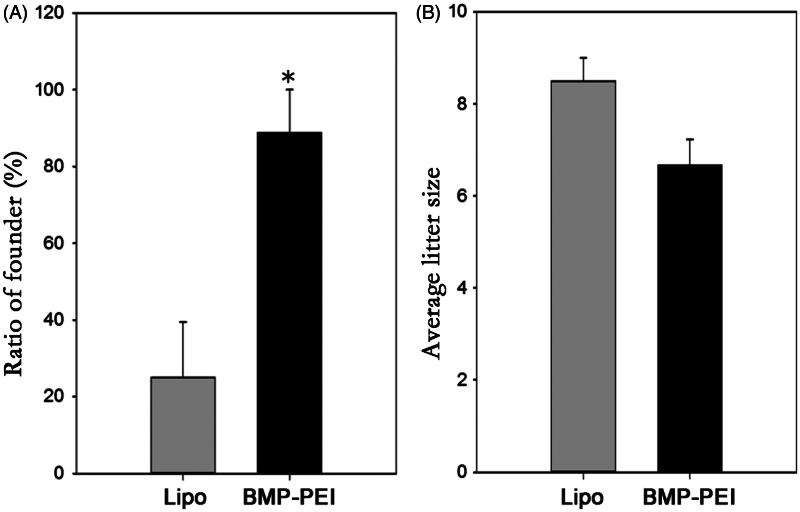
The efficiency of TMGT between BPD and Lipo-DNA groups. (A) The ratios of founder mice obtained from different treatments were significantly different from each other, in which founder mice in BPDs were 3 times more than that in Lipo-DNA group. (B) Upon litter size of founders, there was no significant difference between the two groups. * Indicated significant different (*p* < 0.05).

**Figure 5.**
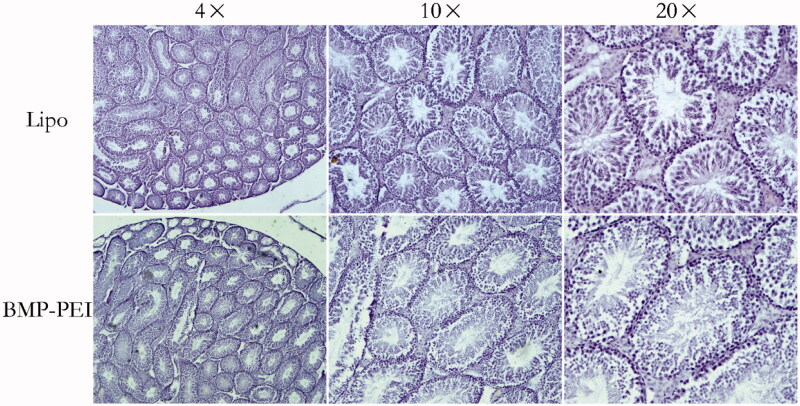
Histochemistry evaluation of the structures of testes that performed TMGT. The results showed that no obvious histological destructions based on seminiferous tubules was observed in the testes of both BPD and Lipo-DNA groups.

By examine the transgenic positive offspring obtained from founder mice with PCR, the ratio of founder obtained from BPDs versus Lipo-DNAs was confirmed. As a result, the ratio of founders obtained from injection of BPDs is significantly higher than that from Lipo-DNAs (88% versus 25%, *p* < 0.05) (Figure 4A). Besides, after supervised the reproduction of the heterozygous, the average litter size between the two groups was proved to be similar to each other (Figure 4B). Last, the histochemistry observation assay showed that there were no obvious damages after the testes of mice were performed with TMGT in this study (Figure 5). Therefore, the results from this study confirmed the hypothesis that BPDs improves transgene efficiency.

## Discussion

In this study, although only few mice were injected with BPDs into their testes, a relatively satisfied output was obtained. As a result, as many as 11 of all 13 injected mice were founders, the production efficiency of which is 3 times more than that of Lipo-DNA group (4 out of 11 mice). The efficiency of producing heterozygous transgene mice from founders was also greatly different between BPDs and the control. Another advantage of BPDs over Lipo-DNAs was its less toxicity to the sperm motility. According to our previous study, BPDs could enhance gene expression of the foreign plasmid in mouse skeleton muscles (Xiang et al., 2007). Altogether, we have showed that BMPs could be an excellent nanoparticle that can assist foreign DNAs transgene in mice and have less toxicity to host cells. Besides, after carefully evaluating the economic and labor factors that influence transgene efficiency between TMGT and traditional transgene production protocols, the authors conclude that TMGT through applying BPDs helps to improve transgene efficiency in several aspects (Table 1). This method may be especially suitable for individual laboratories which lack experienced technicians and necessary equipment to produce transgene mice through pronuclear injection method.

**Table 1.** Comparison of the efficacy of producing founder mice between traditional transgene mice production method and TMGT method.

Items	Pronuclear injection	Intra-testicular injection
Transgenic efficiency (founder%)	About 5–7%^a^	88%
Total mice required	>50	<10
Time for obtaining founders	>2 months	<2 months
Time consumed for operation	>1 week	Half a day
Equipment requirement	Strict and expensive	Rough and cheep
Technicians training	Strict and expensive	Rough and cheep
Labor requirement	High	Low
Costs	Much high	Low

^a^The data came from our lab.

The difficulty of transferring plasmid DNA into the nucleus of a cell has long been a major problem in the successful production of non-viral transgenic animals (Villemejane & Mir, 2009; Kim et al., 2010; Campos et al., 2011a,b). For instance, although a single injection of circular plasmid DNA encapsulated within a liposome was sufficient for transfection of sperm cells (Sato et al., 1999), liposome-mediated transgene seemed to be less effective (4% founder obtained) (Yonezawa et al., 2001). According to the study of He et al., a repeated injection of GFP cDNA into the testis at multiple sites resulted in 41% transgenic founder mice and 37% transgenic positive mice in the first generation (He et al., 2006). However, Sato and Nakamura indicated that repeated injections (3 and 6 repeated injections, 3 days apart) were not critical for introducing high copy numbers of DNA into offspring (Sato & Nakamura, 2004). Furthermore, although DNA-DMSO complex could produce as many as 61%, 55%, and 80% of transgenic founders in mouse and/or rabbit through testes injection, DMSO-containing solution induces testicular degeneration and reduces vascularization around seminiferous tubules (Shen et al., 2006; Amaral et al., 2011). Notably, the efficiency of producing transgenic founder mice through one injection procedure performed in this study by applying BMPs was 88%, which was higher than existed reports.

According to previous studies, magnetic nanoparticles bound to exogenous DNA localized either within or on the plasma membrane, whereas most liposome-bound exogenous DNA localized on the plasma membrane (Scherer et al., 2002). With the help of a magnetic force, BMP may accelerate the accumulation of complexes on the surface of cells and improve nuclear uptake of magnetic particles in the process of magnetofection (Tang et al., 2012). This is especially in agree with our study where the clusters of BMP complex were presented inside and outside of membrane of nuclear in spermatogenic cells of the founders’ testes. It indicates that under the magnetic force, although BMP itself could not improve the DNA integration efficiency, BMPs with natural bio-membrane may provide more opportunity for the DNA to get across the membranes of host cells and reach the nuclear, which increases the possibility of foreign DNAs to integrate to the chromosomes and therefore achieve high efficiency (Xiang et al., 2007).

As has been reported, the 25 kDa PEI combines a strong DNA compaction capacity with an intrinsic endosomolytic activity, namely “proton sponge effect”, for transporting DNAs toward the nucleus (Read et al., 2010; Ma et al., 2011). PEI is capable of condensing DNA into nano-size polyplexes and improves gene delivery efficiency (Felgner et al., 1997). However, the non-biodegradable C-C or C-N bonds within its structure induce accumulative cytotoxicity *in vitro* and *in vivo* (Chollet et al., 2002; Lu et al., 2006; Xiang et al., 2007; Wang et al., 2012). And such cationic polymers may interact with serum proteins resulting in their rapid clearance from the bloodstream, which make it not permissible for use in human. One effective way to diminish the toxicity of PEIs is to reduce the positive charges of PEI (Dash et al., 1999; Chollet et al., 2002; Patnaik et al., 2010). Magnetosomes are attractive carriers of therapeutic drugs and genes for medicines for a long time (Tang et al., 2012). Whenever BMPs conjugating to the PEIs, the positive charges of PEI can be counteracted by the negative charges on the membrane of BMPs (Fischer et al., 1999). Our previous study approved that the BPDs may have no observable influence on the viability of cells in contrast to the cytotoxicity of PEI (Xiang et al., 2007). Additionally, DNases present in the seminal fluid can degrade exogenous DNA molecules. Li et al. approved that BMPs-PEI complexes could bind DNA and provide protection from DNase degradation (Xiang et al., 2007). Similarly, Kim et al. supplied additional proofs that magnetic nanoparticles is better than liposomes in resisting the activity of DNase I from digesting exogenous DNA binding to sperm (Kim et al., 2010). Therefore, BMP may contribute to reduce the toxicity of PEI in our study and promotes transgene.

Although this study obtained proofs of BPDs improved transgenic efficiency in inbred mice strains, it is necessary to verify the effectiveness as well as the applicability among other laboratory animals, such as miniature pigs, nonhuman primates, and large livestock. Recently, a preliminary study of us showed that BPDs was able to deliver foreign DNAs successfully into the testes of sheep (2 out of 4), as were judged from the ejaculated semen 1 month after surgery (data not shown). However, large animals have considerably big gonads, which make it difficult to adjust the volume of solution buffer as well as the concentration of final BPDs. Additional studies also should concentrate on evaluate if BPDs could assist gene modification via nuclease-based gene targeting, including CRISPR/Cas9 as well as NgAgo–gDNA systems (Jinek et al., 2012; Mali et al., 2013; Pauwels et al., 2014; Gao et al., 2016; Vassena et al., 2016).

In conclusion, BPDs applied in this study improve TMGT transgene efficiency, which could be a promising strategy for conveying foreign genes into the testes and achieve transgene effectively.
